# MiR-145 inhibits cell migration and increases paclitaxel chemosensitivity in prostate cancer cells

**DOI:** 10.22038/IJBMS.2023.70878.15397

**Published:** 2023

**Authors:** Maryam Tohidast, Neda Memari, Mohammad Amini, Seyed Samad Hosseini, Asiyeh Jebelli, Mohammad Amin Doustvandi, Behzad Baradaran, Ahad Mokhtarzadeh

**Affiliations:** 1 Department of Biological Science, Faculty of Basic Science, Higher Education Institute of Rab-Rashid, Tabriz, Iran; 2 Immunology Research Center, Tabriz University of Medical Sciences, Tabriz, Iran; 3 Clinical Research Development Unit of Tabriz Valiasr Hospital, Tabriz University of Medical Sciences, Tabriz, Iran; # These authors contributed eqully to this work

**Keywords:** Apoptosis, Chemotherapy, miR-145, Paclitaxel, Prostate cancer

## Abstract

**Objective(s):**

Prostate cancer (PC) is one of the most commonly diagnosed malignancies among men worldwide. Paclitaxel is a chemotherapeutic agent widely used to treat different types of cancer. Recent studies revealed miRNAs control various genes that influence the regulation of many biological and pathological processes such as the formation and development of cancer, chemotherapy resistance, etc.

**Materials and Methods:**

Between three PC cell lines (PC3, DU-145, LNCAP), PC3 showed the lowest miR-145 expression and was chosen for experiments. PC3 cells were treated with paclitaxel and miR-145 separately or in combination. To measure the cell viability, migratory capacity, autophagy, cell cycle progression, and apoptosis induction, the MTT assay, wound-healing assay, and Annexin V/PI apoptosis assay were used, respectively. Moreover, quantitative real-time PCR (qRT-PCR) was employed to measure the expression level of genes involved in apoptosis, migration, and stemness properties.

**Results:**

Obtained results illustrated that miR-145 transfection could enhance the sensitivity of PC3 cells to paclitaxel and increase paclitaxel-induced apoptosis by modulating the expression of related genes, including *Caspase-3, Caspase-9, Bax,* and *Bcl-2*. Also, results showed combination therapy increased cell cycle arrest at the sub-G1 phase. miR-145 and paclitaxel cooperatively reduced migration ability and related-metastatic and stemness gene expression, including *MMP-2, MMP-9, CD44,* and *SOX-2*. In addition, combination therapy can suppress *MDR1* expression.

**Conclusion:**

These results confirmed that miR-145 combined with paclitaxel cooperatively could inhibit cell proliferation and migration and increase the chemosensitivity of PC3 cells compared to mono treatment. So, miR-145 combination therapy may be used as a promising approach for PC treatment.

## Introduction

Prostate cancer (PC) is the second most frequent malignancy in men worldwide and also the second cause of death in the US, counting 33,330 new deaths and 191,930 new PC cases in 2020 (1). The incidence and mortality rates of PC worldwide are associated with increasing age, and the highest occurrence is diagnosed in elderly men. PC incidence rates among African-American men are higher than among White men, and their mortality is almost twice of White men (2). The etiology of PC is the subject of many studies, but it remains less known compared to other types of common cancers. The well-established PC risk factors are advanced age, genetic factors, family history, and ethnicity. Other factors positively related to PC include diet (increased consumption of high-fat foods, lower intake of vegetables, fruits, and vitamins), obesity and physical inactivity, hyperglycemia, inflammation, infections, and environmental exposure to ionizing radiation or chemicals (3). Currently, there are several treatment options for advanced PC, such as chemotherapy, hormone therapy, radium-223, immunotherapy, etc. (4). Chemotherapy has been studied as a therapy strategy for PC since the early 1980s. Docetaxel and Cabazitaxel were approved to treat metastatic castration-resistant PC, and now they are standard care for late-stage disease. Paclitaxel, one of the most widely used natural anticancer agents, is used in adjuvant combination chemotherapy for patients with high-risk localized PC (5). While other tubulin-binding anticancer agents prevent tubulin assembly into microtubules, paclitaxel promotes tubulin assembly into microtubules and suppresses the dissociation of microtubules. It leads to blocking cell cycle progression, ultimately preventing mitosis and inhibiting the growth of cancer cells (6, 7). MicroRNAs (miRNAs) are a class of small non-coding RNAs with 18–25 nucleotide lengths that regulate gene expression post-transcriptionally. miRNAs control the expression of their target genes via inhibiting mRNA translation or degrading mRNA transcripts. Dysregulation of miRNA expression is correlated with cancer initiation, progression, metastasis, and drug resistance. Especially, miRNAs have a critical role in response to environmental stresses, including starvation, hypoxia, DNA damage, and oxidative stress involved in cancer (8-10). miRNAs are potential biomarkers for cancer screening. Since every miRNA can interact with components of several cellular pathways, thus, alterations in the expression of some miRNAs may imply a dysregulation of various cellular processes conforming to the complexity of cancer. miRNA-based diagnostics represent a promising field for early cancer detection methods (11-14). miR-145 acts as a tumor suppressor that targets different tumor-specific genes, consequently affecting related signaling pathways. This miRNA regulates tumor growth, metastasis, invasion, and sensitivity to chemotherapeutic drugs, and it has a vital role in tumor angiogenesis and cancer stem cell proliferation. miR-145 shows tumor suppressor activity in various tumors, such as prostate, colon, bladder, esophageal, and ovarian cancers (15). Drug resistance is the leading cause of therapeutic failure and death of cancer patients. Cumulative evidence has demonstrated that miR-145 is associated with chemotherapy resistance, introducing it as a promising biomarker for drug resistance (16). Given the essential role of miR-145 in cancer cells, in our study, we tried to combine paclitaxel and miR-145 as a novel cancer therapy approach in PC. Our results indicated that paclitaxel and miR-145 cooperatively could reduce cell viability and migration and enhance the rate of apoptosis induction and autophagy. In addition, paclitaxel and miR-145 combination more effectively up-regulated apoptosis-related genes, including *Bax, Caspase-3, and Caspase-9,* and down-regulated metastatic and stemness-related genes including *MMP-2*,* MMP-9*, *SOX-2*, and* CD44*, suggesting the combination of paclitaxel and miR-145 as a promising therapeutic approach for PC treatment. 

## Materials and Methods

***Cell culture and cell line selection***

The human PC cell lines, including LNCAP, DU145, and PC3, were received from Pasture Institute (Tehran, Iran). All cells were kept in a moist atmosphere with 5% CO_2_ and cultured in a standard RPMI-1640 medium containing 1% Penicillin/Streptomycin (Sigma-Aldrich, USA) and 10% fetal bovine serum (Gibco). The expression level of miR-145 was assessed in three PC cell lines by qRT-PCR. 

***microRNA transfection***

PC cells were first dissolved in an electroporation buffer, 1×10^6^ cells were transferred to a 500 µl cuvette, and different doses of miR-145 (10, 20, and 40 pmol) were added. Transfection of miRNA was performed according to the recommended protocol and employing Gene Pulser Xcell electroporation (BioRad). 1×10^4^/well of transfected cells were cultured into 96-well plates and incubated for 48 hr. Finally, the effect of miR-145 relative expression on cell vitality was measured using an MTT assay. The one dose with the highest effect was chosen for the following experiments.

***MTT assay***

To assess whether miR-145 sensitizes PC3 cells to paclitaxel treatment, an MTT assay was performed. The cells were sorted into two groups; one was left untransfected, another was transfected with miR-145, and cells were cultured in a 96-well plate. After 24 hr of incubation, different doses of paclitaxel (1 to 100 μg/ml) were prepared, and cells were treated. After 24 hr, the media was removed, 50 μl of prepared MTT solution with concentration of 2 mg/ml was added to each well, then cells were incubated for 4 hr at 37 ^°^C. Afterward, the MTT solution was exchanged with 200 μl of DMSO. After 45 min, the optical density of wells was measured at 570-630 nm by utilizing an ELISA reader (Sunrise, Tecan, Switzerland). All tests were conducted in triplicate.

***RNA extraction, cDNA synthesis, and qRT-PCR***

The total RNA of cells was extracted utilizing TRIzol reagent (South Korea) according to the instructions. The concentration and the quality of isolated RNAs were assessed by NanoDrop (Thermo Scientific, USA). miRCURY Lna Universal cDNA Synthesis Kit was applied for complementary DNA (cDNA) synthesis. The expression levels of miR-145,* Bcl-2*,* Bax*, 

*Caspase-9*,* Caspase-3*,* MMP-9*,* MMP-2*,* CD44*, *MDR1*, and *SOX-2* were evaluated by qRT-PCR reaction using SYBR Green PCR Master Mix (South Korea). *U6* and *GAPDH* genes were used as standardized internal references to normalize the expression of miRNA and target genes, respectively. Each reaction was independently performed three times, and the relative expression levels of genes were measured by the 2^−ΔΔCt^ method. The primer pair sequences are addressed in Table 1.

***Annexin V/ Propidium Iodide (PI) apoptosis assay***

Apoptosis was estimated using the annexin-V and propidium iodide (PI) Kit. The experiments were subdivided into four groups: three groups were treated with miR-145, paclitaxel, combined miR-145/paclitaxel, and the last one was the control group. The cells were digested with trypsin, then suspended in binding buffer (100 μl), and after that, treated with annexin V-FITC (5 μl) and PI (5 μl) and incubated for 15 min in a dark place. Finally, the apoptosis rates of cells were measured by MACS Quant Flow-cytometry, and data were analyzed with the FlowJo software package. 

***Autophagy***

The simultaneous effect of miR-145 and paclitaxel on autophagy activation was assessed utilizing monodansylcadaverine (MDC) staining. Briefly, PC3 cells (2 × 10^5^ cells/well) were cultured in a six-well plate, and after treatment and incubation, the cells were rinsed with PBS, incubated at 37 ^°^C in 500 μl MDC (50 μM) for 10 min. Then, the cells were rinsed with PBS, harvested, and subjected to flow-cytometry. Finally, the data were analyzed with the FlowJo software package.

***Cell cycle analysis***

The cell cycle progression between treatment groups was detected utilizing DAPI dye and analyzed by flow-cytometry. 2×10^5^ cells were cultured in a six-well plate. After treatment and incubation, cells were digested by trypsinization, rinsed with PBS, fixed in 75% ice-cold ethanol, and incubated at -20 ^°^C overnight. The cells were rinsed and exposed to PBS containing RNase A (1%) and incubated for 30 min. After that, the cells were rewashed, stained with DAPI solution (0.1% Triton X100 and 0.1% DAPI), and incubated for 30 min in a dark place. Ultimately, cell cycle status was assessed by flow-cytometry. Obtained data were analyzed using FlowJo software.

***Wound healing assay***

To assess cell migration ability, a wound-healing assay was used. PC3 cells were transfected with miR-145 and cultured in a 24-well plate. When cells reached 70-80% confluence, a wound area was made via a sterile yellow pipette tip across the cell monolayer. The media of each well were removed and treated with paclitaxel. Eventually, images of wells were captured using an inverted light microscope (Optika, Italy) at 0, 12, 24, and 48 hr after treatment.

***Statistical analysis***

All data in the experiments were performed in triplicate and analyzed statistically utilizing the GraphPad Prism 8.0 software package. Data were expressed as the means±standard deviation (SD). Student’s t-test was used to assess statistical differences between the two groups. Also, a one-way analysis of variance (ANOVA) was performed to compare multiple groups. A *P*-value<0.05 was considered to illustrate a statistically significant difference.

## Results

***miR-145 expression level in PC cell lines***

**Figure 1:**
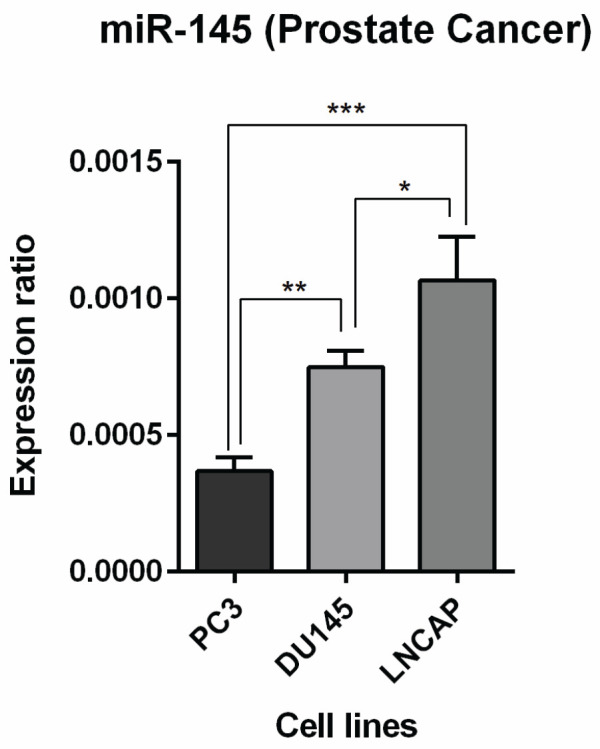
MiR-145 expression level was assessed in three PC cell lines, including DU145, LNCAP, and PC3, relative to each other (*** *P<*0.001)

To choose the suitable cell line, first, the expression level of miR-145 was evaluated in DU145, LNCAP, and PC3 cell lines employing qRT-PCR. As shown in Figure 1, among all three cell lines, LNCAP has the highest expression of miR-145, and the PC3 cell line represented the lowest expression level of miR-145 (*P*<0.001). So, according to the obtained results, the PC3 cell line was chosen to continue the experiments.

***Evaluation of optimum dose and efficient transfection of miR-145 into PC3 cells***

**Figure 2:**
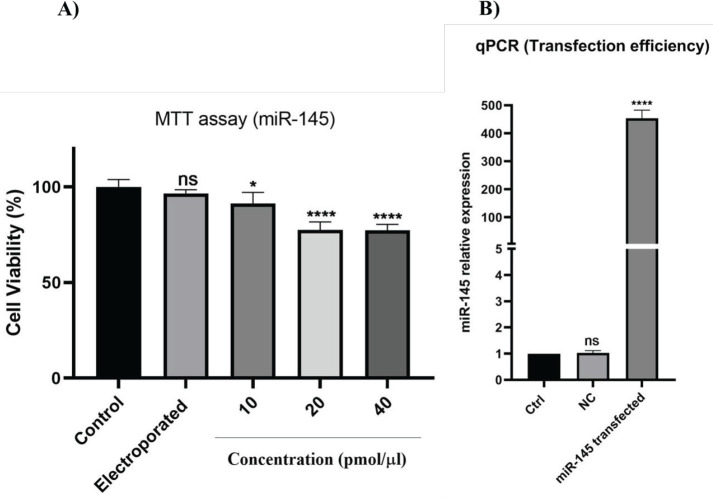
A) To determine the optimum transfection dose of miR-145, PC3 cells were transfected by different concentrations of miR-145 (10, 20, and 40 pmol), and cytotoxicity was measured using MTT assay (**** *P<*0.0001)

The optimum dose of miR-145 transfection to cells was determined by the MTT assay. Various doses of miR-145 were prepared (10, 20, and 40 pmol), and PC3 cells were transfected. After 48 hr, cell viability and proliferation were measured via MTT assay. Our results indicated that 20 and 40 pmol of miR-145 remarkably reduced cell viability (*P*<0.0001), so the 20 pmol dose was chosen for the transfection of PC3 cells (Figure 2A). To evaluate the efficiency of miRNA transfection by electrophoretic apparatus, cells were transfected with negative control miRNA and miR-145 (20 pmol) and analyzed with qRT-PCR. The results illustrated that after transfection, the expression level of miR-145 significantly increased compared to the negative control group (*P*<0.0001) (Figure 2B).

***miR-145 cooperatively enhanced paclitaxel-induced cytotoxicity***

**Figure 3:**
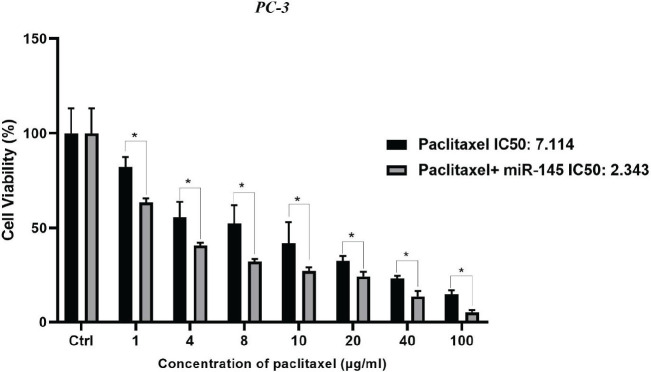
Cytotoxic effect of paclitaxel alone or combined with miR-145 was assessed in various doses by MTT

To investigate whether miR-145 treatment could increase the sensitivity of the PC3 cells to paclitaxel, an MTT assay was performed. As reflected in Figure. 3, treatment of paclitaxel alone could significantly diminish the viability rate in a dose-dependent manner. Also, the combination of miR-145/paclitaxel considerably reduced the cell viability compared to groups treated with paclitaxel alone. Obtained results showed that miR-145 transfection increased the sensitivity of PC3 cells to paclitaxel. As seen in Figure 3, the IC50 of paclitaxel in the alone and combination groups was 7.114 μg/ml and 2.343 μg/ml, respectively, remarkably decreased (*P*<0.0001).

Furthermore, we evaluated the synergistic cytotoxic effect of paclitaxel and miR-145 on PC3 cells by calculating the combination index (CI) using the formula described by Chou and Talalay (17, 18):

CI=(%Viability Combination)/(%Viability miR-145 * %Viability)

CI values less than 1, equal to 1, and greater than 1 indicate synergism, additivity, and antagonism in the combined drug action, respectively. As depicted in Table 2, the results obtained demonstrate that various concentrations of paclitaxel and miR-145 exhibited a synergistic cytotoxic effect on PC3 cells (CI < 1). In other words, the combination of paclitaxel and miR-145 effectively reduced PC3 cell viability to a greater extent than individual treatments.

***miR-145 and paclitaxel combination effect on cell apoptosis***

**Figure 4:**
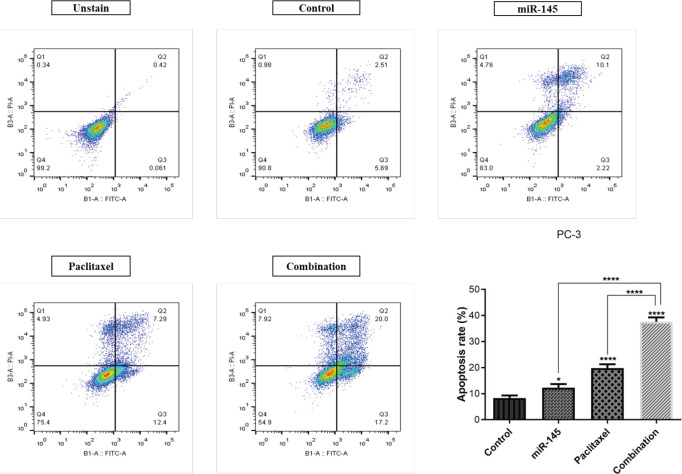
The apoptosis induction was evaluated by Annexin V-FITC/PI staining in treatment groups, include unstain, control, miR-145, paclitaxel, and combination (**** *P<*0.0001)

**Figure 5:**
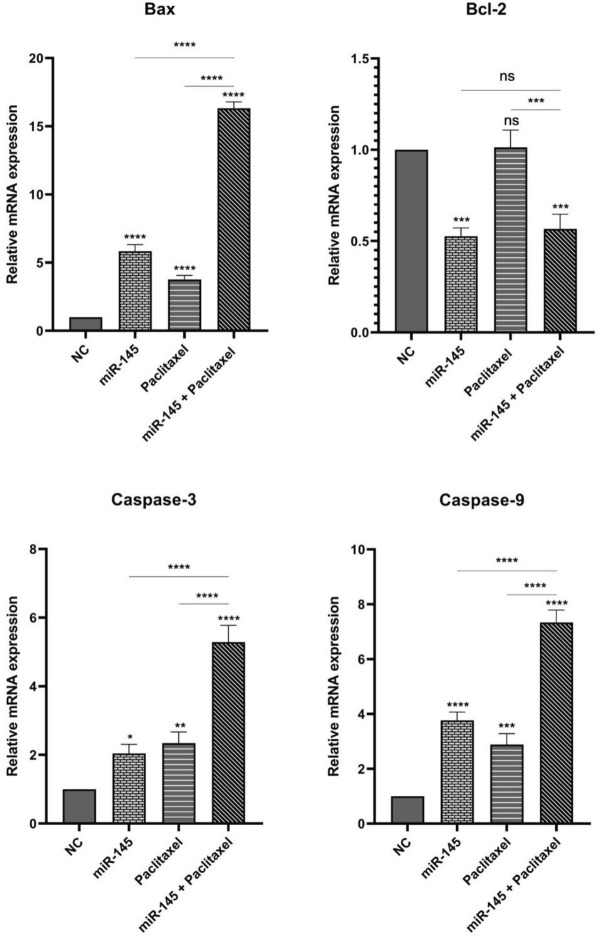
Expression levels of Bcl-2, Bax, Caspase-3, and Caspase-9 mRNA were measured using qRT-PCR (**** *P<*0.0001) (ns: non-significant)

The combination effect of miR-145/paclitaxel on apoptosis induction was evaluated by applying Annexin V/PI staining. Our results indicated that miR-145 and paclitaxel considerably enhanced the apoptosis rate in PC3 cells from 8.2% to 12.32% and 19.69%, respectively. In addition, in the combination group, the percentage of apoptotic cells increased to 37.2% compared to the control group (Figure 4). Furthermore, to evaluate the expression of apoptosis-related genes, the qRT-PCR technique was employed. Our results demonstrated that miR-145 and paclitaxel raised the expression level of *Bax*, *Caspase-3*, and *Caspase-9* as pro-apoptotic genes. Also, in the combination group, expression of these genes significantly increased compared to alone treated groups. miR-145 reduced the *Bcl-2* expression as a pro-survival gene, but paclitaxel did not have a noticeable effect on *Bcl-2* expression (ns). In combination groups expression level of *Bcl-2* significantly reduced (*P*<0.001) (Figure 5).

***MiR-145 and paclitaxel effect on autophagy induction***

**Figure 6:**
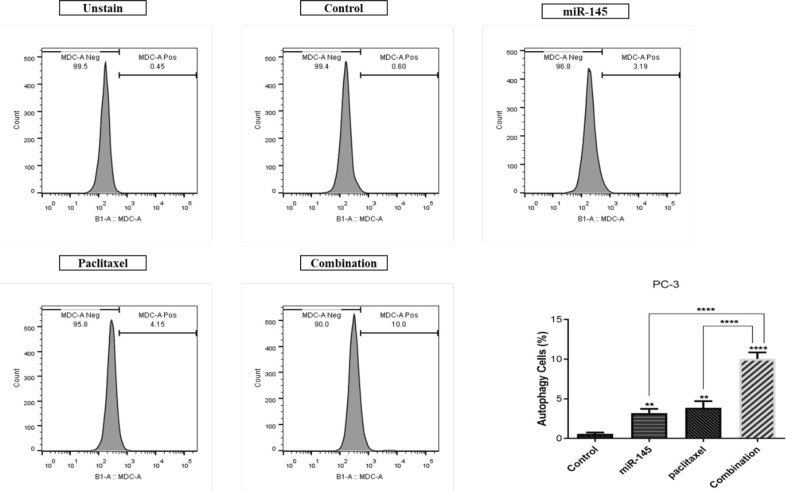
Evaluation of autophagy induction between treatment groups in PC3 cells (**** *P<*0.0001)

This study evaluated autophagy induction in the treatment groups as a mechanism involving cell death using flow-cytometry. Our results demonstrated that treating cells with miR-145 and paclitaxel alone enhanced the rate of autophagy to 3.19% and 4.15%, respectively, compared to the control (0.60%). Moreover, the miR-145/paclitaxel combination could increase autophagy induction (10.0%) more effectively than separate groups compared to the control (Figure 6).

***Combination of paclitaxel and miR-145 increased PC3 cell cycle progression at G2/M and sub-G1***

**Figure 7:**
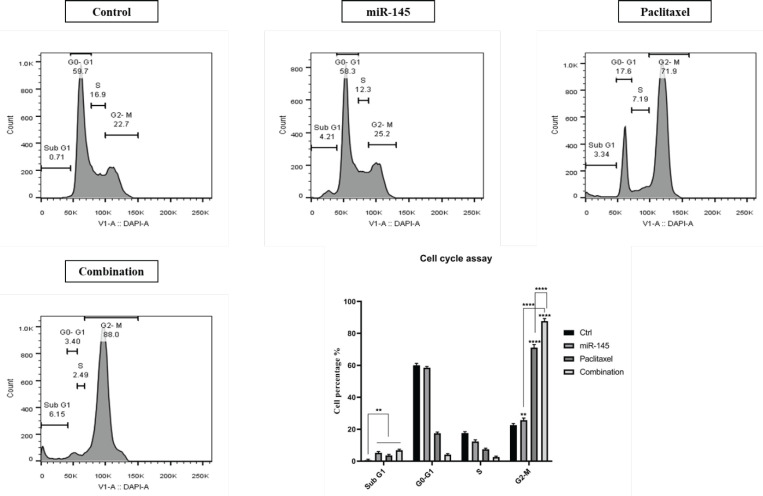
Effect of paclitaxel and miR-145, alone or combination, on the cell cycle status (**** *P<*0.0001)

Cell cycle assay was performed employing flow-cytometry and DAPI staining. Obtained results demonstrated that paclitaxel and miR-145 could significantly enhance cell population in the G2/M phase of the cell cycle in PC3 cells. In cells treated with paclitaxel, the population of the G2/M cells increased from 22.7% to 71.9%. miR-145 enhanced the cell population at the G2/M phase from 22.7% to 25.2%. Importantly, the combination treatment increased cell cycle arrest at the G2/M remarkably more than the separate groups (88%). Additionally, paclitaxel and miR-145 triggered cell cycle arrest at the sub-G1 phase from 0.71% to 3.34% and 4.21%, respectively. Also, in the combination group, this rate increased to 6.15% (Figure 7).

***MiR-145 and paclitaxel inhibited PC3 cell migration***

**Figure 8:**
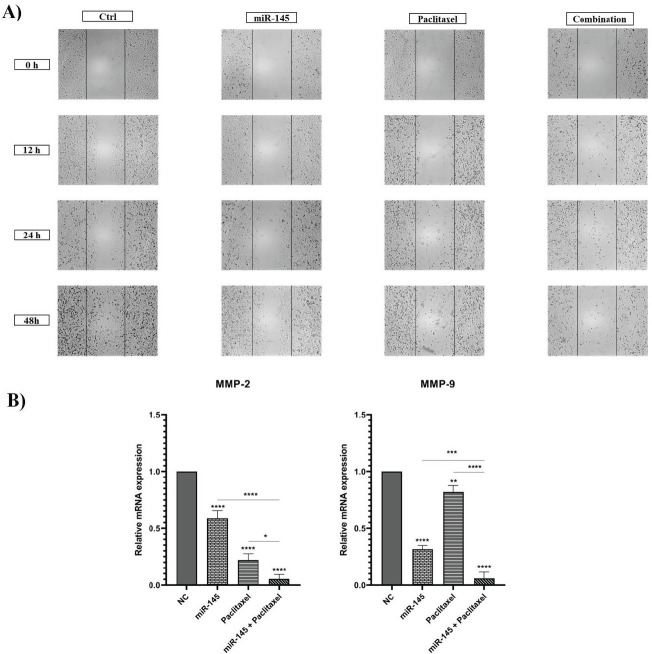
A) Effect of paclitaxel and miR-145 on cell migration was evaluated utilizing the wound-healing assay which was pictured at different times (0, 12, 24, and 48 hr). B) Expression levels of MMP-2 and MMP-9 were assessed using qRT-PCR (**** *P<*0.0001)

Since cancer cell migration is an essential step of metastasis, the effect of paclitaxel and miR-145, alone or in combination, on the migration of PC3 cells was assessed by wound healing assay. According to Figure 8A, the results displayed a notable decline in the migration of the miR-145-treated group compared to the control group. Also, paclitaxel treatment significantly decreased PC3 cell migration. Also, combination therapy reduced the migration ability of cells more effectively than alone treated groups. To validate the results of the wound healing assay, the expression levels of metastasis-related genes, including *MMP-2* and *MMP-9* were evaluated by qRT-PCR. Results displayed that treated cells with paclitaxel and miR-145 alone could remarkably reduce the expression level of these genes. This reduction was considerably more in the combination group than separately treated groups (*P*<0.0001) (Figure 8B). These results suggest that miR-145 and paclitaxel might cooperatively suppress PC3 cell migration by down-regulation of metastasis-related genes.

***Paclitaxel and miR-145 additionally decrease prostate cancer stemness properties***

miR-145 and paclitaxel effects on cancer stemness properties were evaluated using the qRT-PCR. We measured the expression level of stemness-related genes, including *CD44* and 

**Figure 9:**
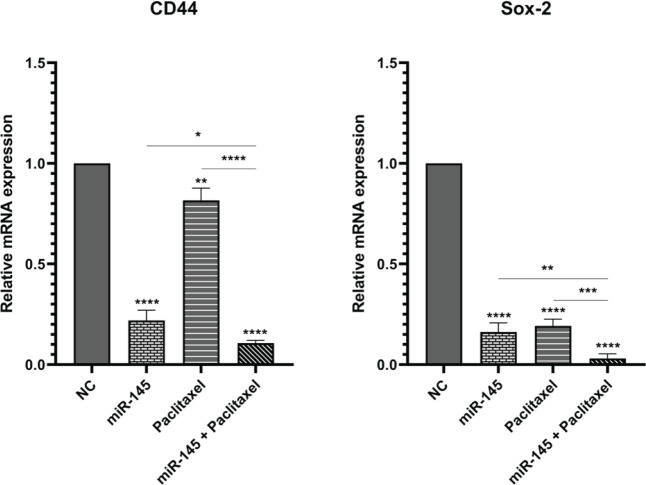
Expression levels of CD44 and SOX-2 were assessed using qRT-PCR (**** *P<*0.0001)

*SOX-2*. Obtained results displayed that miR-145 and paclitaxel individually could significantly reduce *CD44* and *SOX-2* expression compared to control cells. In addition, miR-145 and paclitaxel could simultaneously decrease the expression of *CD44* and *SOX-2* more effectively than separate treatment groups (Figure 9). 

***miR-145 suppressed expression of the MDR1 gene in PC3 cells***

**Figure 10:**
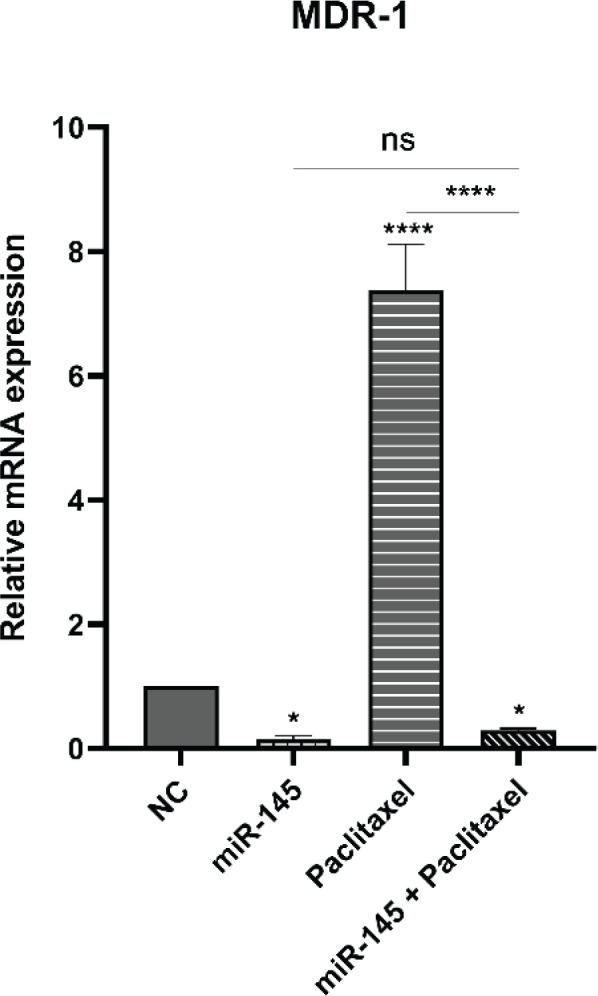
Expression levels of multi-drug resistance (MDR1) were assessed using qRT-PCR (****P<0.0001) (ns: non-significant)

The MDR1 (multi-drug resistance) Protein 1 is a member of the ATP-binding cassette (ABC) transporters family, responsible for transporting various substances, including toxic compounds and medical drugs, out of cells. Its role in chemotherapy resistance has made the activity of the MDR1 gene a subject of extensive research in cancer cells (19). Our study demonstrates that treating PC3 cells with paclitaxel significantly increased the expression of the *MDR1* gene. However, when combined with miR-145, the expression of *MDR1* was notably reduced (****P<0.0001) (Figure 10). The difference in *MDR1* expression level between miR-145 and the combination group is not significant. These findings indicate that miR-145 can effectively suppress the overexpression of *MDR1* induced by paclitaxel in PC3 cells. 

**Table 1:** Sequences of gene primers used in this study for qRT-PCR assay

Gene	Forward and reverse	Sequences
*GAPDH*	F	5ˊ-CAAGATCATCAGCAATGCCT-3ˊ
	R	5ˊ-GCCATCACGCCACAGTTTCC-3ˊ
*U6*	F	5ˊ-CTTCGGCAGCACATATACTAAAATTGG-3ˊ
	R	5ˊ-TCATCCTTGCGCAGGGG-3ˊ
*Bax*	F	5′‐GACTCCCCCCGAGAGGTCTT‐3′
	R	5′‐ACAGGGCCTTGAGCACCAGTT‐3′
*Bcl-2*	F	5ˊ-CTGTGGATGACTGAGTACCTG-3ˊ
	R	5ˊ-GAGACAGCCAGGAGAAATCA-3ˊ
*Caspase-9*	F	5′-CCGGAATCCTGCTTGGGTATC-3′
	R	5′-CATCGGTGCATTTGGCATGTA-3′
*Caspase-3*	F	5′-GTGGAACTGACGATGATATGGC-3′
	R	5′-CGCAAAGTGACTGGATGAACC-3′
*CD44*	F	5′-CCAGAAGGAACAGTGGTTTGGC-3′
	R	5′-ACTGTCCTCTGGGCTTGGTGTT-3′
*MMP-9*	F	5′-GGTTCTTCTGCGCTACTGCTG-3′
	R	5′-GTCGTAGGGCTGCTGGAAGG-3′
*MMP-2*	F	5′-AGCGAGTGGATGCCGCCTTTAA-3′
	R	5′-CATTCCAGGCATCTGCGATGAG-3′
*SOX-2*	F	5′-GCTACAGCATGATGCAGGACCA-3′
	R	5′-TCTGCGAGCTGGTCATGGAGTT-3′
*MDR1*	F	5′-GCTACAGCATGATGCAGGACCA-3′
	R	5′-CTGCGAGCTGGTCATGGAGTT-3′

B) miR-145 expression level was measured employing the qRT-PCR technique after transfection in PC3 cells compared to the control (**** *P<*0.0001) (ns: non-significant)

**Table 2:** Inhibitory concentration values and combination indexes. The results show that different concentrations of paclitaxel and miR-145 display a synergistic cytotoxic effect on PC3 cells (Mean CDI < 1)

Groups	Concentration							Mean CDI
	1	4	8	10	20	40	100	
Paclitaxel	82.20%	55.69%	52.24%	41.93%	32.57%	22.91%	14.83%	
miR-145	77.58%	77.58%	77.58%	77.58%	77.58%	77.58%	77.58%	
Paclitaxel+ miR-145	63.35%	41.52%	32.03%	26.94%	24.27%	13.62%	5.21%	
CI= %Viability Combination / %Viability miR-145 * %Viability	0.993472437	0.961049346	0.790188474	0.828309771	0.960437719	0.766502863	0.453238464	0.821885582

## Discussion

PC is the most frequent noncutaneous malignancy affecting men. The majority of PCs are diagnosed in elderly men. As the population ages, the incidence of PC increases. Presently, about 10 million men live with a PC diagnosis, and approximately 700,000 of them are living with metastatic disease. PC management continues to progress rapidly, with fundamental advances in perceiving the genomic landscape of both primary and metastatic PC (20, 21). For decades, paclitaxel has been consumed for cancer treatment, and it is one of the most successful chemotherapy agents in the clinic and financially. Chemotherapy resistance is the main problem in cancer treatment. Long-term use of paclitaxel might increase the chance of drug resistance, leading to chemotherapy failure. The underlying mechanisms of chemotherapy resistance are still not well understood. Some studies indicated chemotherapy resistance was associated with particular miRNAs (22). Various studies have reported the role of miRNAs in paclitaxel chemoresistance. Zhou *et al*. showed that in breast cancer, miR-125b was up-regulated in taxol-resistant cells, which significantly inhibited taxol-induced cytotoxicity and apoptosis and subsequently increased taxol resistance in cancer cells. Suppression of miR-125b restored taxol sensitivity and overcame miR-125-mediated taxol resistance (23). Researchers demonstrated that in gastric cancer, miR34a could enhance the chemotherapeutic efficacy of paclitaxel. Furthermore, their results showed that the transcription factor E2F 5 (E2F5), a critical oncogenic protein, is a candidate direct target of miRNA-34a (24). miR-145 plays a crucial role in therapeutic resistance acquisition, demonstrated by accumulating evidence. Also, miR-145 participates in various biological processes of cancers via regulating target genes or signaling, such as proliferation, differentiation, apoptosis, tumorigenesis, metastasis, and angiogenesis. Recent evidence has suggested miR-145 as a new therapeutic target and predictive marker of response that could further raise the efficacy of chemotherapy (25). Previous research investigated the role of miR-145 in paclitaxel-resistant ovarian cancer patients. It was found that the expression level of miR-145 was decreased, and the expression of Sp1 and Cdk6 was up-regulated. Restoration of miR-145 in ovarian cancer cells can enhance chemosensitivity and increase the accumulation of antineoplastic drugs and G1 cell cycle arrest (26).

According to the results of the MTT assay, pretreatment of PC3 cells by miR-145 could enhance the chemosensitivity of cells to paclitaxel treatment. It means the combination of miR-145 and paclitaxel reduced cell proliferation and viability more than paclitaxel treatment alone. These results are consistent with the findings of the previous studies. A study reported the up-regulation of miR-145 using miRNA overexpressed vectors could improve the chemosensitivity of glioblastoma cells to demethoxycurcumin by targeting the SOX-2-Wnt/β-catenin axis (27). Zheng *et al*. indicated miR-145 sensitized esophageal squamous cell carcinoma (ESCC) to cisplatin and facilitated cisplatin-induced apoptosis via directly inhibiting the PI3K/AKT signaling pathway (28). 

Moreover, in the present study, additional analysis utilizing flow-cytometry demonstrated that treatment of PC3 cells with miR-145 and paclitaxel alone significantly increased the apoptosis induction rate compared to the control group. Also, in the combination group, the rate of apoptosis was remarkably more than in separately treated groups. The expression levels of apoptosis-related genes were measured utilizing the qRT-PCR technique for more investigation. Bcl2 family proteins regulate the intrinsic mitochondrial apoptosis pathway in response to cellular stresses, including cytokine withdrawal, growth-factor deprivation, DNA damage, Ca^++^-flux, and

γ-irradiation. Still, they can contribute to cell death triggered via tumor necrosis factor family members. Deregulation of Bcl2 family proteins is a common feature of human cancer diseases and a cause of therapy resistance. Plenty of studies revealed the pro-and anti-apoptotic role of Bcl2 family proteins in tumor pathogenesis and mediating anticancer agents (29, 30). Caspase family members play crucial roles in apoptosis initiation and execution. Caspases are subdivided into two groups: the initiator (apical) caspases, including Caspase-2, 8, 9, and 10, and the executioner (effector) caspases, including Caspase-3, 6, and 7. Many conventional anti-cancer therapies trigger apoptosis to kill the cancer cells by engaging the caspases family indirectly (31). Our results illustrated considerable up-regulation of *Bax, Caspase-3*, and *Caspase-9* in the combination group compared with groups treated separately and the control group. Also, results indicated miR-145 could significantly decrease the Bcl-2 expression, but paclitaxel did not have a significant effect (ns). In the combination group, the expression level of *Bcl-2* was remarkably reduced compared to the paclitaxel group. In agreement with our results, Ren *et al*. demonstrated treatment of canine mammary gland tumor cells with paclitaxel could suppress proliferation and induce apoptosis. Also, paclitaxel treatment could increase the expression of *Bax* and *Caspase-3* expression levels (32). Previous studies have indicated that miR-145 induces apoptosis in cancer cells (33, 34). A study reported that miR-145 inhibits the proliferation and induces apoptosis of NSCLC cells by up-regulation of the *Bax* and *Caspase-3* expression and down-regulation of *Bcl-2* (35). 

Autophagy is a highly conserved catabolic mechanism that allows the degradation of damaged intracellular components and metabolite recycling. Autophagy plays an essential role in the response of cancer cells to therapy as cancer therapies often inflict damage and stress on cells to induce cell death (36, 37). Therefore, considering its significance, in this study, we investigated miR-145 and paclitaxel involvement in the autophagy induction in PC3 cells. Obtained results from MDC staining indicated that treating cells with miR-145 and paclitaxel alone could enhance the autophagy rate compared to control cells. Also, the results indicated that the combination of miR-145 and paclitaxel could increase the autophagy rate compared to separate treatment groups. Consistent with our results, a previous study demonstrated that paclitaxel induces autophagy in breast cancer cells by regulating the ERK1/2 signaling pathway (38). Moreover, overexpression of miR-145 in neuroblastoma cells was shown to inhibit tumorigenesis through autophagy induction (39). 

The current study assessed the effect of miR-145 and paclitaxel on cell-cycle progression. Results showed both miR-145 and paclitaxel separately could enhance the rate of PC3 cells arrested at sub-G1 and G2/M phases compared to control cells. Also, in the combination group, the rate of cell cycle arrest was significantly more than alone treated groups. It was shown that paclitaxel could enhance sub-G1 and G2/M cell cycle arrest (40). Research indicated that miR-145, in bladder cancer cells, increases the cell population of the G2/M arrested cells (41). Also, Law *et al*. reported that re-expression of miR-145 in hepatocellular carcinoma cells could trigger G2/M cell cycle arrest (42). Invasion and migration of cancer cells are the main features of tumor biology, which are the leading causes of death in cancer patients. Malignant cells disseminate and migrate through multiple alternative mechanisms, such as mesenchymal cell migration, collective cell migration, and amoeboid cell migration. This plasticity in motility patterns gives the ability to cancer cells to disseminate further and therefore reduces anti-metastasis therapy’s efficiency (43). The wound-healing assay was performed to explore miR-145 and paclitaxel effects on the migration ability of PC3 cells. Results illustrated that separately treating cells with paclitaxel and miR-145 could inhibit migration. Also, the combination of miR-145/paclitaxel could suppress migration more than alone treated groups. In addition, the expression of main metastasis-related genes, including *MMP-2, MMP-9, *and stemness-related genes, including *CD44* and *SOX-2 *were measured by qRT-PCR. Matrix metalloproteinases (MMPs) are a group of endopeptidases that participate in the initial stages of cancer invasion. They can disrupt all extracellular matrix (ECM) molecules and basement membrane (BM). MMPs are involved in the release and activation of different cytokines, chemokines, growth factors, cytoskeletal proteins, and adhesion molecules, which allow them to contribute to various physiological events, such as wound healing, inflammation, embryogenesis angiogenesis, and bone remodeling. Participation of MMPs in cancer progression makes them attractive targets for cancer therapy (44). CD44 is a complex transmembrane glycoprotein that exists in numerous molecular forms. CD44 participates in numerous physiological processes, and its dysregulation and aberrant expression correlate to tumor initiation and progression. Many studies reported CD44 as a biomarker of cancer stem cells (CSCs) and promoter of epithelial-mesenchymal transition. Moreover, CD44 is associated with regulating essential signaling pathways that modulate invasion, proliferation, metastasis, and therapy resistance in cancer cells (45). The transcription factor SOX-2 is vital for embryonic development and has a crucial role in stem cell self-renewal, homeostasis, and reprogramming. SOX-2 dysregulation is associated with plenty of cancer types, and it has positive effects on cancer cell features such as proliferation, invasion, migration, and metastasis. Therefore, SOX-2 is an attractive anticancer target (46, 47). Our results indicated that miR-145 and paclitaxel alone could significantly reduce the expression levels of *MMP-2, MMP-9, CD44,* and *SOX-2* compared to control cells. Also, results illustrated that combination therapy could remarkably decrease the expression levels of these genes more effectively than the separate treatment groups. A previous study demonstrated paclitaxel could inhibit invasion and migration in gastric cancer cells through down-regulation of *MMP-9* and cyclooxygenase2 expression (48). It was shown that miR-145 could inhibit the migration of breast cancer cells by regulating TGF-β1 expression (49). Research indicated miR-145 could considerably inhibit the invasion and proliferation of ovarian cancer cells by down-regulating *MMP-2* and *MMP-9* (50). Zeng *et al*. reported that miR-145 suppresses tumor growth and chemo-resistance by targeting *CD44* mRNA in gastric cancer (51). Also, miR-145 could significantly decrease the expression level of *SOX-2* and inhibit proliferation and migration in PC cells (52). 

Many cancer types initially respond to chemotherapy but ultimately develop resistance due to various factors. These factors include cellular reprogramming, oncogenic stimulation, increased expression of multi-drug resistance (MDR) genes leading to drug efflux, metabolic changes that deactivate and inhibit drugs, altered DNA damage repair mechanisms, epithelial-mesenchymal transition (EMT), evasion of programmed cell death, inherent cell heterogeneity, metabolic reprogramming, epigenetic changes, or a combination of these mechanisms (53). MDR1 represents a promising target for combating chemoresistance and improving cancer therapy. Several studies have provided compelling evidence indicating that dysregulation of miRNAs plays a crucial role in either promoting resistance or enhancing sensitivity to different standard chemotherapy drugs across various types of cancer (54, 55). The findings from our study demonstrated a significant increase in MDR1 expression in PC3 cells following treatment with paclitaxel. However, in the combination group, the expression of *MDR1* was significantly reduced. These results indicate that miR-145 plays a role in suppressing *MDR1* expression. Importantly, this suggests that by suppressing *MDR1*, miR-145 can enhance the chemosensitivity and improve the effectiveness of paclitaxel treatment on PC3 cells.

## Conclusion

Generally, our findings established that combination therapy of paclitaxel and miR-145 could effectively diminish PC3 proliferation and induce cell apoptosis by regulating *Bax/Bcl-2* and *Caspase-3/9* expression levels. Our results illustrated that combination therapy enhanced autophagy induction and cell cycle arrest at sub-G1 and G2/M phases. Moreover, combination therapy inhibited cell migration and stemness properties by reducing *MMP-2, MMP-9, CD44,* and *SOX-2*, and* MDR1* gene expression levels. Finally, these findings suggested that the combination therapy of paclitaxel and miR-145 might be introduced as a promising therapeutic approach for developing novel methods to treat PC.

## Authors’ Contributions

M T and N M performed the majority of experiments and data analysis. M A, SS H, A J, and MA D contributed to carrying out the experiments and interpreted the results. SS H wrote the manuscript and helped with experiments. B B revised the manuscript critically for important intellectual content. A M designed and conducted the project.

## Availability of data and material

All data generated in this study are included in the manuscript.

## Code availability

Not applicable.

## Ethical approval

The study was approved by the ethical committee of Tabriz University of Medical Sciences, Tabriz, Iran.

## Consent to participate

Not applicable.

## Consent for publication

Not applicable. 

## Conflicts of Interest

The authors declare no conflicts of interest.

## References

[1] Golubnitschaja O, Kubatka P, Mazurakova A, Samec M, Alajati A, Giordano FA (2022). Systemic effects reflected in specific biomarker patterns are instrumental for the paradigm change in prostate cancer management: A strategic paper. Cancers.

[2] Panigrahi GK, Praharaj PP, Kittaka H, Mridha AR, Black OM, Singh R (2019). Exosome proteomic analyses identify inflammatory phenotype and novel biomarkers in African American prostate cancer patients. Cancer Med.

[3] Rawla P (2019). Epidemiology of prostate cancer. World J Oncol.

[4] Sumanasuriya S, De Bono J (2018). Treatment of advanced prostate cancer-A review of current therapies and future promise. Cold Spring Harb Perspect Med.

[5] Quinn D, Sandler H, Horvath L, Goldkorn A, Eastham J (2017). The evolution of chemotherapy for the treatment of prostate cancer. Ann Oncol.

[6] Khanna C, Rosenberg M, Vail D (2015). A review of paclitaxel and novel formulations including those suitable for use in dogs. J Vet Intern Med.

[7] Zhu L, Chen L (2019). Progress in research on paclitaxel and tumor immunotherapy. Cell Mol Biol Lett.

[8] Otmani K, Lewalle P (2021). Tumor suppressor miRNA in cancer cells and the tumor microenvironment: Mechanism of deregulation and clinical implications. Front Oncol.

[9] Annese T, Tamma R, De Giorgis M, Ribatti D (2020). microRNAs biogenesis, functions and role in tumor angiogenesis. Front Oncol.

[10] Jebelli A, Oroojalian F, Fathi F, Mokhtarzadeh A, de la Guardia M (2020). Recent advances in surface plasmon resonance biosensors for microRNAs detection. Biosens Bioelectron.

[11] Vanacore D, Boccellino M, Rossetti S, Cavaliere C, D’Aniello C, Di Franco R (2017). Micrornas in prostate cancer: An overview. Oncotarget.

[12] Jahanafrooz Z, Motamed N, Rinner B, Mokhtarzadeh A, Baradaran B (2018). Silibinin to improve cancer therapeutic, as an apoptotic inducer, autophagy modulator, cell cycle inhibitor, and microRNAs regulator. Life Sci.

[13] Asl ER, Amini M, Najafi S, Mansoori B, Mokhtarzadeh A, Mohammadi A (2021). Interplay between MAPK/ERK signaling pathway and MicroRNAs: A crucial mechanism regulating cancer cell metabolism and tumor progression. Life Sci.

[14] Rezaei T, Amini M, Hashemi ZS, Mansoori B, Rezaei S, Karami H (2020). microRNA-181 serves as a dual-role regulator in the development of human cancers. Free Radic Biol Med.

[15] Ye D, Shen Z, Zhou S (2019). Function of microRNA-145 and mechanisms underlying its role in malignant tumor diagnosis and treatment. Cancer Manag Res.

[16] Mansoori B, Mohammadi A, Davudian S, Shirjang S, Baradaran B (2017). The different mechanisms of cancer drug resistance: A brief review. Adv Pharm Bull.

[17] Chou T-C (2006). Theoretical basis, experimental design, and computerized simulation of synergism and antagonism in drug combination studies. Pharmacol Rev.

[18] Tun JO, Salvador-Reyes LA, Velarde MC, Saito N, Suwanborirux K, Concepcion GP (2019). Synergistic cytotoxicity of renieramycin M and doxorubicin in MCF-7 breast cancer cells. Mar Drugs.

[19] Bossennec M, Di Roio A, Caux C, Ménétrier-Caux C (2018). MDR1 in immunity: Friend or foe?. Oncoimmunology.

[20] Dunn MW, Kazer MW (2011). Prostate cancer overview. Semin Oncol Nurs.

[21] Sandhu S, Moore CM, Chiong E, Beltran H, Bristow RG, Williams SG (2021). Prostate cancer. Lancet.

[22] Ma Y, Yu S, Ni S, Zhang B, Kung ACF, Gao J (2021). Targeting strategies for enhancing paclitaxel specificity in chemotherapy. Front Cell Dev Biol.

[23] Zhou M, Liu Z, Zhao Y, Ding Y, Liu H, Xi Y (2010). MicroRNA-125b confers the resistance of breast cancer cells to paclitaxel through suppression of pro-apoptotic Bcl-2 antagonist killer 1 (Bak1) expression. J Biol Chem.

[24] Li L, Wu C, Zhao Y (2017). miRNA-34a enhances the sensitivity of gastric cancer cells to treatment with paclitaxel by targeting E2F5. Oncol Lett.

[25] Xu W, Hua Y, Deng F, Wang D, Wu Y, Zhang W (2020). MiR-145 in cancer therapy resistance and sensitivity: A comprehensive review. Cancer Sci.

[26] Zhu X, Li Y, Xie C, Yin X, Liu Y, Cao Y (2014). MiR-145 sensitizes ovarian cancer cells to paclitaxel by targeting Sp1 and Cdk6. Int J Cancer.

[27] Qian C, Wang B, Zou Y, Zhang Y, Hu X, Sun W (2019). MicroRNA 145 enhances chemosensitivity of glioblastoma stem cells to demethoxycurcumin. Cancer Manag Res.

[28] Zheng T-L, Li D-P, He Z-F, Zhao S (2019). MiR-145 sensitizes esophageal squamous cell carcinoma to cisplatin through directly inhibiting PI3K/AKT signaling pathway. Cancer Cell Int.

[29] Frenzel A, Grespi F, Chmelewskij W, Villunger A (2009). Bcl2 family proteins in carcinogenesis and the treatment of cancer. Apoptosis.

[30] Campbell KJ, Tait SW (2018). Targeting BCL-2 regulated apoptosis in cancer. Open Biol.

[31] Boice A, Bouchier-Hayes L (2020). Targeting apoptotic caspases in cancer. Biochim Biophys Acta Mol Cell Res.

[32] Ren X, Zhao B, Chang H, Xiao M, Wu Y, Liu Y (2018). Paclitaxel suppresses proliferation and induces apoptosis through regulation of ROS and the AKT/MAPK signaling pathway in canine mammary gland tumor cells. Mol Med Rep.

[33] Zheng M, Wu Z, Wu A, Huang Z, He N, Xie X (2016). MiR-145 promotes TNF-α-induced apoptosis by facilitating the formation of RIP1-FADDcaspase-8 complex in triple-negative breast cancer. Tumor Biol.

[34] Xin Z, Tong Z, Tan J, Liu C (2021). MicroRNA-145-5p aggravates cell apoptosis and oxidative stress in tongue squamous cell carcinoma. Exp Ther Med.

[35] Pan Y, Ye C, Tian Q, Yan S, Zeng X, Xiao C (2018). miR-145 suppresses the proliferation, invasion and migration of NSCLC cells by regulating the BAX/BCL-2 ratio and the caspase-3 cascade. Oncol Lett.

[36] Mulcahy Levy JM, Thorburn A (2020). Autophagy in cancer: Moving from understanding mechanism to improving therapy responses in patients. Cell Death Differ.

[37] Grácio D, Magro F, Lima RT, Máximo V (2017). An overview on the role of autophagy in cancer therapy. Hematol Med Oncol.

[38] Lee Y, Na J, Lee MS, Cha EY, Sul JY, Park JB (2018). Combination of pristimerin and paclitaxel additively induces autophagy in human breast cancer cells via ERK1/2 regulation. Mol Med Rep.

[39] Kim KW, Qiao J, Kim JY, Park K, Chung DH (2020). Overexpression of microRNA-145 inhibits tumorigenesis through autophagy in chemotherapy and radiation resistant neuroblastoma cells. Oncoscience.

[40] Shu C-H, Yang W, Shih Y-L, Kuo M-L, Huang T-S (1997). Cell cycle G2/M arrest and activation of cyclin-dependent kinases associated with low-dose paclitaxel-induced sub-G1 apoptosis. Apoptosis.

[41] Matsushita R, Yoshino H, Enokida H, Goto Y, Miyamoto K, Yonemori M (2016). Regulation of UHRF1 by dual-strand tumor-suppressor microRNA-145 (miR-145-5p and miR-145-3p): Inhibition of bladder cancer cell aggressiveness. Oncotarget.

[42] Law PT-Y, Ching AK-K, Chan AW-H, Wong QW-L, Wong C-K, To K-F (2012). MiR-145 modulates multiple components of the insulin-like growth factor pathway in hepatocellular carcinoma. Carcinogenesis.

[43] Wu J-s, Jiang J, Chen B-j, Wang K, Tang Y-l, Liang X-h (2021). Plasticity of cancer cell invasion: Patterns and mechanisms. Transl Oncol.

[44] Gonzalez-Avila G, Sommer B, Mendoza-Posada DA, Ramos C, Garcia-Hernandez AA, Falfan-Valencia R (2019). Matrix metalloproteinases participation in the metastatic process and their diagnostic and therapeutic applications in cancer. Crit Rev Oncol Hematol.

[45] Xu H, Niu M, Yuan X, Wu K, Liu A (2020). CD44 as a tumor biomarker and therapeutic target. Exp Hematol Oncol.

[46] Novak D, Hüser L, Elton JJ, Umansky V, Altevogt P, Utikal J (2020). SOX2 in development and cancer biology. Semin Cancer Biol.

[47] Zhang S, Xiong X, Sun Y (2020). Functional characterization of SOX2 as an anticancer target. Signal Transduct Target Ther.

[48] Sun K, Tang XH, Xie YK (2015). Paclitaxel combined with harmine inhibits the migration and invasion of gastric cancer cells through downregulation of cyclooxygenase2 expression. Oncol Lett.

[49] Ding Y, Zhang C, Zhang J, Zhang N, Li T, Fang J (2017). miR-145 inhibits proliferation and migration of breast cancer cells by directly or indirectly regulating TGF-β1 expression. Int J Oncol.

[50] Wang M, Zhang S (2021). MiR-145 on the proliferation of ovarian cancer cells by regulating the expression of MMP-2/MMP-9. Cell Mol Biol.

[51] Zeng J-F, Ma X-Q, Wang L-P, Wang W (2017). MicroRNA-145 exerts tumor-suppressive and chemo-resistance lowering effects by targeting CD44 in gastric cancer. World J Gastroenterol.

[52] Ozen M, Karatas OF, Gulluoglu S, Bayrak OF, Sevli S, Guzel E (2015). Overexpression of miR-145-5p inhibits proliferation of prostate cancer cells and reduces SOX2 expression. Cancer Invest.

[53] Vaidya FU, Sufiyan Chhipa A, Mishra V, Gupta VK, Rawat SG, Kumar A (2022). Molecular and cellular paradigms of multidrug resistance in cancer. Cancer Rep.

[54] Lampis A, Hahne JC, Hedayat S, Valeri N (2020). MicroRNAs as mediators of drug resistance mechanisms. Curr Opin Pharmacol.

[55] Safaei S, Amini M, Najjary S, Mokhtarzadeh A, Bolandi N, Saeedi H (2022). MiR-200c increases the sensitivity of breast cancer cells to Doxorubicin through downregulating MDR1 gene. Exp Mol Pathol.

